# Lithium intoxication and its implications, pertaining to a clinical case

**DOI:** 10.1192/j.eurpsy.2021.1659

**Published:** 2021-08-13

**Authors:** A. Quintão, H. Simião

**Affiliations:** Psychiatry Department, Ocidental Lisbon Hospital Center, Lisboa, Portugal

**Keywords:** lithium, Intoxication

## Abstract

**Introduction:**

Lithium is the most effective maintenance drug in Bipolar Disorder (BD), although it has a narrow therapeutic index, between 0.6 and 1.5 mEq/L; recommended doses for maintenance are 0.6-1.2 mEq/L.

**Objectives:**

To describe a clinical case of lithium intoxication and discuss relevant literature.

**Methods:**

Clinical examination of a patient and her medical records; non-systematic PubMed review on “lithium intoxication”.

**Results:**

A 73-year-old woman, diagnosed with BD, stabilized on lithium monotherapy for twenty-five years, was admitted to the Emergency Room (ER) with nausea, lethargy, drowsiness, confusion, cough, and fever. A respiratory tract infection is diagnosed, based on clinical presentation, x-ray and blood analysis. Blood tests also revealed a serum lithium concentration of 2.4 mEq/L and impairment of renal function, indicating lithium intoxication; hemodialysis was initiated, with lithium discontinuation. Over weeks, renal function and general state improved, and BD treatment was reinitiated, this time with valproic acid 800mg/day. Two weeks after discharge, she was admitted again at the ER, for an episode starting in the week prior, compatible with a manic episode; olanzapine 10mg was added to the prescription. A week after, the patient is admitted again in the ER, still in a manic episode.

**Conclusions:**

There are no clear indications in the literature about reinitiating lithium on someone who had an intoxication. Given that lithium brings an unparalelled quality of life to BD patients, careful consideration about reintroduction, with close monitoring, should be made, but there is a critical need of more studies and guidelines to orient clinical practice.

**Disclosure:**

No significant relationships.

